# Skyrmions and Spin Waves in Magneto–Ferroelectric Superlattices

**DOI:** 10.3390/e22080862

**Published:** 2020-08-04

**Authors:** Ildus F. Sharafullin, Hung T. Diep

**Affiliations:** 1 Institute of Physics and Technology, Bashkir State University, 32, Validy str, Ufa 450076, Russia; sharafullinif@yandex.ru; 2 Laboratoire de Physique Théorique et Modélisation, CY Cergy Paris Université, CNRS, UMR 8089, 2 Avenue Adolphe Chauvin, CEDEX, 95302 Cergy-Pontoise, France

**Keywords:** superlattice, magneto–ferroelectric coupling, spin waves, skyrmions, phase transition, Green’s function theory, Monte Carlo simulation

## Abstract

We present in this paper the effects of Dzyaloshinskii–Moriya (DM) magneto–electric coupling between ferroelectric and magnetic interface atomic layers in a superlattice formed by alternate magnetic and ferroelectric films. We consider two cases: magnetic and ferroelectric films have the simple cubic lattice and the triangular lattice. In the two cases, magnetic films have Heisenberg spins interacting with each other via an exchange *J* and a DM interaction with the ferroelectric interface. The electrical polarizations of ±1 are assumed for the ferroelectric films. We determine the ground-state (GS) spin configuration in the magnetic film and study the phase transition in each case. In the simple cubic lattice case, in zero field, the GS is periodically non collinear (helical structure) and in an applied field H perpendicular to the layers, it shows the existence of skyrmions at the interface. Using the Green’s function method we study the spin waves (SW) excited in a monolayer and also in a bilayer sandwiched between ferroelectric films, in zero field. We show that the DM interaction strongly affects the long-wave length SW mode. We calculate also the magnetization at low temperatures. We use next Monte Carlo simulations to calculate various physical quantities at finite temperatures such as the critical temperature, the layer magnetization and the layer polarization, as functions of the magneto–electric DM coupling and the applied magnetic field. Phase transition to the disordered phase is studied. In the case of the triangular lattice, we show the formation of skyrmions even in zero field and a skyrmion crystal in an applied field when the interface coupling between the ferroelectric film and the ferromagnetic film is rather strong. The skyrmion crystal is stable in a large region of the external magnetic field. The phase transition is studied.

## 1. Introduction

Magnetic skyrmions are topological configurations of spin vortices [1,2]. Skyrmions and vortices have drawn enormous attention due to their fundamental and practical importance [3,4,5,6,7,8]. Skyrmions hold great promise as a basis for future digital technologies [9,10,11,12,13,14,15,16,17,18]. Information flow in next-generation spintronic devices could be associated with metastable isolated skyrmions guided along magnetic stripes [19,20]. Ferromagnetic skyrmions formed in ferromagnet/ferroelectric or heavy-metal multilayers [5] are generally considered as elements of skyrmionic race-track memory [17,21]. Skyrmions have been experimentally observed, created and manipulated in a number of material systems, including magnetic materials [4,6,7,10,22,23,24,25,26,27,28,29,30], multiferroic materials and superlattices [31,32,33]. Skyrmions have been recently observed in conducting and insulating helimagnets under an applied magnetic field [4,23]. Under an applied magnetic field, the spiral structure due to a Dzyaloshinskii–Moriya (DM) transforms into a skyrmion crystal [34].

The anomalous thermodynamic properties of the paradigmatic frustrated spin-1/2 triangular-lattice Heisenberg magnetic films has remained an open topic of research over decades, both experimentally and theoretically. Shen et al. have demonstrated that the observed unusual spin correlations and thermodynamics can be described by a transverse field Ising model on the triangular lattice with a ferro-multipolar order [35]. Kurumaji et al. have reported the emergence of a Bloch-type skyrmion state in the frustrated centrosymmetric triangular-lattice magnet $Gd_{2}PdSi_{3}$ [36]. They have observed a giant topological Hall response, indicating a field-induced skyrmion phase. Note that the phase diagram of a spin-1/2 Heisenberg antiferromagnet on the triangular lattice with first- and second-neighbor interactions was investigated in Ref. [37]. It was discovered regions of $120^{\circ }$ and collinear stripe order, separated by an intermediate nonmagnetic phase. Remarkably, numerical simulations performed in Ref. [38] revealed two crossover low-temperature and high-temperature scales. It is shown that in the intermediate regime between the low-temperature scale and the higher one, the ’rotonlike’ excitations are activated with a strong chiral component.

Multiferroic materials, or materials with the simultaneously coexistence and coupling of ferroelectric and magnetic orders, have returned to the forefront of condensed matter research. Developing heterostructures and superlattices of multiferroics (two-component ${\left[A/B\right]}_{N}$ superlattice with *A*—magnetic film, *B*—ferroelectric film, *N*—number of repetitions) are attracting increasing interest and provoking extensive research activity due to the broken mirror symmetry at interface between two materials (the Dzyaloshinskii–Moriya interaction can only appear in systems that break bulk inversion symmetry or mirror symmetry). Therefore, superstructures naturally lead to the creation of skyrmions at different interfaces which have a unique dynamics compared to the spin structures created at the interface of the same elements. Enormous attention was paid to studying the non-uniform states in magnetic/ferroelectric superlattices both theoretically [39] and experimentally [40,41,42].

Recently, magnetic/ferroelectric superlattices attract much of attention as magneto–electric (ME) materials [41,42]. Spin waves and skyrmions in “unfrustrated” magnetoelectric superlattices with a Dzyaloshinskii–Moriya (DM) interface coupling have been studied [39].

Note that in Ref. [39], we investigated the effects of Dzyaloshinskii–Moriya (DM) magneto-ferroelectric interaction in an unfrustrated ferromagnetic/ferroelectric superlattice. In zero external magnetic field, we showed that the ground-state spin configuration is periodically non-collinear. Through the use of a two-time Green’s functions, we calculated the spin wave spectrum in a monolayer and in a bilayer sandwiched between two ferroelectric films. We showed that when magnetic field is applied in the direction perpendicular to the plane of layers, the skyrmions are arranged to form a crystalline structure at the interface. In Ref. [43] the effect of the frustration in a superlattice composed of alternating frustrated magnetic and ferroelectric films was investigated. We showed in particular that the frustration gives rise to an enhancement of skyrmions created by the DM interaction at the magneto–electric interface in an external field. In these works, we have considered the magnetic and ferroelectric films with a simple cubic lattice.

The purpose of this paper is to show the main features of our results obtained for a magneto–ferroelectric superlattice where the interface coupling is a DM interaction. The effect of the frustration due to the competing next-nearest-neighbor (NNN) interaction is emphasized. Since the interface coupling with a DM interaction creates the frustration at the interface, it is desirable to consider the case of triangular lattice because this lattice is known to be frustrated when non-collinear spin configurations are present. The purpose of this paper includes thus this so-far-missing case to see the effect of the DM interface coupling on the properties of skyrmions. As it turns out, we find several striking features among which (i) skyrmions are created even without an applied magnetic field, (ii) skyrmions are stable in a large region of magnetic field. These results are important for applications because skyrmions which are magnetic textures can be manipulated by an electric field.

The paper is organized as follows. Section 2 is devoted to the description of our model and the determination of the ground-state spin configuration with and without applied magnetic field. Skyrmion crystal is shown with varying interface parameters. Section 3 shows the results for spin-wave spectrum obtained by the Green’s function method. Results of Monte Carlo simulations for the phase transition in the system is shown in Section 4. These results show that the skyrmion crystal is stable at finite temperatures below a transition temperature $T_{c}$. The case of the triangular lattice is shown in Section 5 and Section 6. A discussion on experimental interest of our model is given in Section 7. Concluding remarks are given in Section 8.

## 2. Model—Ground State

Consider a superlattice composed of alternate magnetic and ferroelectric films. Both have the structure of simple cubic lattice of the same lattice constant, for simplicity. The Hamiltonian of this multiferroic superlattice is expressed as:

(1) $$H=H_{m}+H_{f}+H_{mf}$$

where $H_{m}$ and $H_{f}$ are the Hamiltonians of the magnetic and ferroelectric subsystems, respectively, $H_{mf}$ denotes Hamiltonian of magneto–electric interaction at the interface of two adjacent films. We are interested in the frustrated regime. Therefore we describe the Hamiltonian of the magnetic film with the Heisenberg spin model on a simple cubic lattice as follows:

(2) $$H_{m}=-\sum_{i,j}J_{ij}^{m}S_{i}\cdot S_{j}-\sum_{i,k}J_{ik}^{2m}S_{i}\cdot S_{k}-\sum_{i}H\cdot S_{i}$$

where $S_{i}$ is the spin on the i-th site, H the external magnetic field, $J_{ij}^{m}$ the magnetic interaction between two spins at *i* and *j* sites. We shall take into account both the nearest neighbors (NN) interaction, denoted by $J^{m}$, and the next-nearest neighbor (NNN) interaction denoted by $J^{2m}$. We consider $J^{m}>0$ to be the same everywhere in the magnetic film. To introduce the frustration we shall consider $J^{2m}<0$, namely antiferromagnetic interaction, the same everywhere. The external magnetic field H is applied along the *z*-axis which is perpendicular to the plane of the layers. The interaction of the spins at the interface will be given below.

For the ferroelectric film, we suppose for simplicity that electric polarizations are Ising-like vectors of magnitude 1, pointing in the ±z direction. The Hamiltonian is given by

(3) $$H_{f}=-\sum_{i,j}J_{ij}^{f}P_{i}\cdot P_{j}-\sum_{i,k}J_{ik}^{2f}P_{i}\cdot P_{k}$$

where $P_{i}$ is the polarization on the i-th lattice site, $J_{ij}^{f}$ the interaction parameter between polarizations at sites *i* and *j*. Similar to the magnetic subsystem we will take the same $J_{ij}^{f}=J^{f}>0$ for all NN pairs, and $J_{ij}=J^{2f}<0$ for NNN pairs.

Before introducing the DM interface interaction, let us emphasize that the bulk $J_{1}-J_{2}$ model on the simple cubic lattice has been studied with Heisenberg spins [44] and the Ising model [45] where $J_{1}$ and $J_{2}$ are both antiferromagnetic (<0). The critical value $|J_{2}^{c}\left|=0.25\right|J_{1}|$ above which the bipartite antiferromagnetic ordering changes into a frustrated ordering where a line is with spins up, and its neighboring lines are with spins down. In the case of $J^{m}>0$ (ferro), and $J^{2m}<0$, it is easy to show that the critical value where the ferromagnetic becomes antiferromagnetic is $J_{c}^{2m}=-0.5J^{m}$. Below this value, the antiferromagnetic ordering replaces the ferromagnetic ordering.

**Figure 1:**
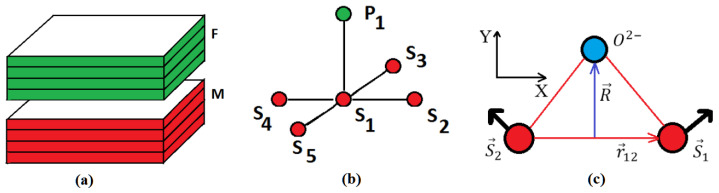
(**a**) Magneto-ferroelectric superlattice composed of alternately a ferroelectric film and a ferromagnetic film. Each of the ferroelectric and ferromagnetic films has *n* atomic layers as shown, (**b**) interfacial coupling between a polarization *P* with 5 spins in a Dzyaloshinskii–Moriya (DM) interaction, (**c**) positions of the spins in the xy plane and the position of non magnetic ion Oxygen, defining the DM vector (see text).

We know that the DM interaction is written as

(4) $$H_{DM}=D_{i,j}\cdot S_{i}\times S_{j}$$

where $S_{i}$ is the spin of the i-th magnetic ion, and $D_{i,j}$ is the Dzyaloshinskii–Moriya vector. The vector $D_{i,j}$ is proportional to the vector product $R\times r_{i,j}$ of the vector R which specifies the displacement of the ligand (for example, oxygen) and the unit vector $r_{i,j}$ along the axis connecting the magnetic ions *i* and *j* (see Figure 1). One then has

(5) $$\begin{array}{ccc} D_{i,j} & = & R\times r_{i,j}\\ D_{j,i} & = & R\times r_{j,i}=-D_{i,j} \end{array}$$

We define thus

(6) $$D_{i,j}=De_{i,j}z$$

where *D* is a constant, z the unit vector on the *z* axis, and $e_{i,j}=-e_{j,i}=1$.

For the magneto–electric interaction at the interface, we choose the interface Hamiltonian following Ref. [39]:

(7) $$H_{mf}=\sum_{i,j,k}J_{i,j}^{mf}e_{i,j}P_{k}\cdot \left[S_{i}\times S_{j}\right]$$

where $P_{k}$ is the polarization at the site *k* of the ferroelectric interface layer, while $S_{i}$ and $S_{j}$ belong to the interface magnetic layer. In this expression $J_{i,j}^{mf}e_{i,j}P_{k}$ plays the role of the DM vector perpendicular to the xy plane, given by Equation (6). When summing the neighboring pairs (i,j), attention should be paid on the signs of $e_{i,j}$ and $S_{i}\times S_{j}$ (see example in Ref. [39]).

Hereafter, we suppose $J_{i,j}^{mf}=J^{mf}$ independent of (i,j).

Since $P_{k}$ is in the *z* direction, i.e., the DM vector is in the *z* direction, in the absence of an applied field the spins in the magnetic films will lie in the xy plane to minimize the interface interaction energy, according to Equation (7).

From Equation (7), we see that the magneto–electric interaction $J^{mf}$ favors a canted (non collinear) spin structure. It competes with the exchange interactions $J^{m}$ and $J^{2m}$ of $H_{m}$ which favor collinear (ferro and antiferro) spin configurations. In ferroelectric films, there is just ferro- or antiferromagnetic ordering due to the Ising nature. Usually the magnetic or ferroelectric exchange interaction is the leading term in the Hamiltonian, so that in many situations the magneto–electric effect is negligible. However, in nanofilms of superlattices the magneto–electric interaction is crucial for the creation of non-collinear long-range spin order. It has been shown that Rashba spin-orbit coupling can lead to a strong DM interaction at the interface [46,47], where the broken inversion symmetry at the interface can change the magnetic states.

Since the polarizations are along the *z* axis, the interface DM interaction is minimum when $S_{i}$ and $S_{j}$ lie in the xy interface plane and perpendicular to each other. However the collinear exchange interactions among the spins will compete with the DM perpendicular configuration. The resulting configuration is non collinear. In the general case where a magnetic field is applied and there is also the frustration, we use the steepest descent method [34,39] to determine the ground state.

**Figure 2:**
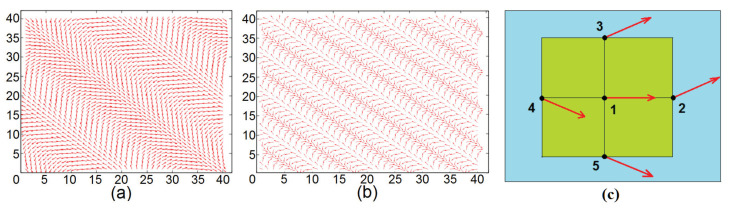
Ground-state (GS) spin configurations for (**a**): $J^{mf}=-0.45$, (**b**): $J^{mf}=-1.2$, with H=0, (**c**): angles between nearest neighbors (NN) are schematically zoomed. See text for comments.

We show in Figure 2 an example where H=0 with no NNN interaction. The magnetic film has a single layer. The GS spin configurations have periodic structures. Several remarks are in order:

(i) Each spin has the same turning angle θ with its NN in both *x* and *y* direction. The schematic zoom in Figure 2c shows that the spins on the same diagonal (spins 1 and 2, spins 3 and 4) are parallel. This explains the structures shown in Figure 2a,b.

(ii) The periodicity of the diagonal parallel lines depends on the value of θ (comparing Figure 2a and Figure 2b). With a large size of *N*, the periodic conditions have no significant effects.

**Figure 3:**
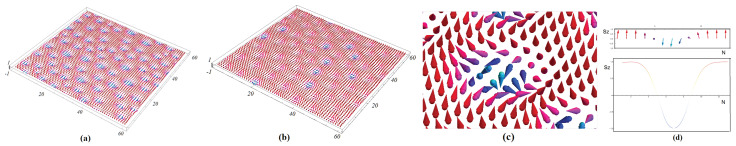
(**a**) 3D view of the GS configuration of the interface for moderate frustration $J^{2m}=J^{2f}=-0.2$. (**b**) 3D view of the GS configuration of the second magnetic layers, (**c**) zoom of a skyrmion on the interface layer: red denotes up spin, four spins with clear blue color are down spin, other colors correspond to spin orientations between the two. The skyrmion is of the Bloch type, (**d**) *z*-components of spins across the skyrmion shown in (**c**). Other parameters: $J^{m}=J^{f}=1$, $J^{mf}=-1.25$ and H=0.25.

Let us show now an example with H≠0 in Figure 3 with a frustration due to NNN interaction.

At this field strength H=0.25, if we increase the frustration, for example $J^{2m}=J^{2f}=-0.3$, then the skyrmion structure is enhanced: we can observe a clear 3D skyrmion crystal structure not only in the interface layer but also in the interior layers (not shown).

## 3. Spin Waves

Here let us show theoretically spin-waves (SW) excited in the magnetic film in zero field, in some simple cases of the simple cubic lattice. The method we employ is the Green’s function technique for non collinear spin configurations which has been shown to be efficient for studying low-*T* properties of quantum spin systems such as helimagnets [48] and systems with a DM interaction [49]. This technique is rather lengthy to describe here. The reader is referred to these papers for mathematical details.

Let us show here the resulting spin-wave energy in the case of the NN interaction only (the case including the NNN interaction which is more complicated will be subject of a future study):

(8) $$\begin{array}{ccc} E & = & \pm \sqrt{\left(A+B)(A-B\right)} \end{array}$$

(9) $$\begin{array}{ccc} A & = & -J^{m}\left[8<S^{z}>\cos\theta -4<S^{z}>\gamma \left(\cos\theta +1\right)\right]\\ & & -4D\sin\theta <S^{z}>\gamma +8D\sin\theta <S^{z}> \end{array}$$

(10) $$\begin{array}{ccc} B & = & 4J^{m}<S^{z}>\gamma \left(\cos\theta -1\right)-4D\sin\theta <S^{z}>\gamma \end{array}$$

where $\gamma =(\cos k_{x}a+\cos k_{y}a)/2$, $k_{x}$ and $k_{y}$ being the wave-vector components in the xy planes, *a* the lattice constant, θ the angle between two NN spins given by $\theta =\arctan(-\frac{2J^{mf}}{J^{m}})$. $<S^{z}>$ is the statistical average of the *z* spin-component. Other parameters $J^{m}$ and *D* have been defined in Equations (2) and (6).

**Figure 4:**
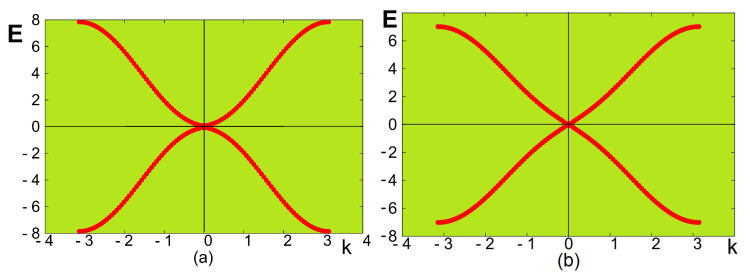
Spin-wave energy E(k) versus *k* ($k\equiv k_{x}=k_{z}$) for (**a**) θ=0.3 radian and (**b**) θ=1 for a monolayer at T=0. See text for comments.

We show in Figure 4 the spin-wave energy *E* versus the wave vector $k=k_{x}=k_{y}$, for weak and strong values of the DM interaction strength *D*. For the weak value of θ (Figure 4a), namely weak $J^{mf}$, the long-wave length mode energy (k→0) is proportional to $k^{2}$ as in ferromagnets, while for strong θ (Figure 4b), *E* is proportional to *k* as in antiferromagnets. The effect of the DM interaction is thus very strong on the spin-wave behavior.

The case of a bilayer as well as the effects of other parameters on the spin-wave spectrum are shown in Ref. [39].

## 4. Phase Transition

This section concerns the simple cubic lattice considered above. We have seen the skyrmion crystal in Figure 3 at T=0. Shall this structure survive at finite *T*? To answer this question we have performed Monte Carlo simulations using the Metropolis algorithm [50]. We have to define an order parameter for the skyrmion crystal. In complicated spin orderings such as spin glasses, we have to follow each individual spin during the time. If it is frozen then its time average is not zero. This is called Edwards–Anderson order parameter for spin glasses [51].

For the magnetic films, the definition of an order parameter for a skyrmion crystal is not obvious. Taking advantage of the fact that we know the GS, we define the order parameter as the projection of an actual spin configuration at a given *T* on its GS and we take the time average. This order parameter of layer *n* is thus defined as

(11) $$M_{m}\left(n\right)=\frac{1}{N^{2}\left(t_{a}-t_{0}\right)}\sum_{i\in n}\left|\sum_{t=t_{0}}^{t_{a}}S_{i}\left(T,t\right)\cdot S_{i}^{0}\left(T=0\right)\right|$$

where $S_{i}\left(T,t\right)$ is the i-th spin at the time *t*, at temperature *T*, and $S_{i}\left(T=0\right)$ is its state in the GS. The order parameter $M_{m}\left(n\right)$ is close to 1 at very low *T* where each spin is only weakly deviated from its state in the GS. $M_{m}\left(n\right)$ is zero when every spin strongly fluctuates in the paramagnetic state.

For the ferroelectric films, the order parameter $M_{f}\left(n\right)$ of layer *n* is defined as the magnetization

(12) $$M_{f}\left(n\right)=\frac{1}{N^{2}}\langle |\sum_{i\in n}P_{i}^{z}|\rangle$$

where 〈…〉 denotes the time average. The total order parameters $M_{m}$ and $M_{f}$ are the sum of the layer order parameters, namely $M_{m}=\sum_{n}M_{m}\left(n\right)$ and $M_{f}=\sum_{n}M_{f}\left(n\right)$.

**Figure 5:**
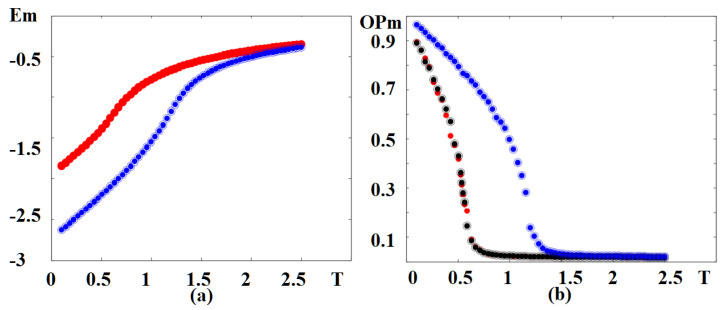
(**a**) Energy of the magnetic films versus temperature *T* for $\left(J^{2m}=J^{2f}=-0.4\right)$ (red), coinciding with the curve for $\left(J^{2m}=-0.4,J^{2f}=0\right)$ (black, hidden behind the red curve). Blue curve is for $\left(J^{2m}=0,J^{2f}=-0.4\right)$; (**b**) order parameter of the magnetic films versus temperature *T* for $\left(J^{2m}=J^{2f}=-0.4\right)$ (red), $\left(J^{2m}=-0.4,J^{2f}=0\right)$ (black), $\left(J^{2m}=0,J^{2f}=-0.4\right)$ (blue). Other used parameters: $J^{mf}=-1.25$, H=0.25.

In Figure 5 we show the magnetic energy and magnetic order parameter versus temperature in an external magnetic field, for various sets of NNN interactions. Note that the phase transition occurs at the energy curvature changes, namely at the maximum of the specific heat. The red curve in Figure 5a is for both sets $\left(J^{2m}=J^{2f}=-0.4\right)$ and $\left(J^{2m}=-0.4,J^{2f}=0\right)$. The change of curvature takes place at $T_{c}^{m}≃0.60$. It means that the ferroelectric frustration does not affect the magnetic skyrmion transition at such a strong magnetic frustration $\left(J^{2m}=-0.4\right)$. For $\left(J^{2m}=0,J^{2f}=-0.4\right)$, namely no magnetic frustration, the transition takes place at a much higher temperature $T_{c}^{m}≃1.25$. The magnetic order parameters shown in Figure 5b confirm the skyrmion transition temperatures seen by the curvature change of the energy in Figure 5a.

## 5. The Case of Triangular Lattice

We consider the same Hamiltonian as described by equations from (1) to (7). The only difference is from now the lattice is triangular. For simplicity, we do not take into account the NNN magnetic interaction in Equation (2).

### 5.1. Ground State in Zero Field

Let us determine the ground-state spin configurations in zero field in the case of a magnetic monolayer, sandwiched between two ferroelectric films. The ferromagnetic exchange interaction among the spins will compete with the perpendicular DM term. The resulting configuration is non-collinear. We note that the ferroelectric film has always polarizations along the *z* axis due to their Ising character, even when the interface interaction is turned on.

**Figure 6:**
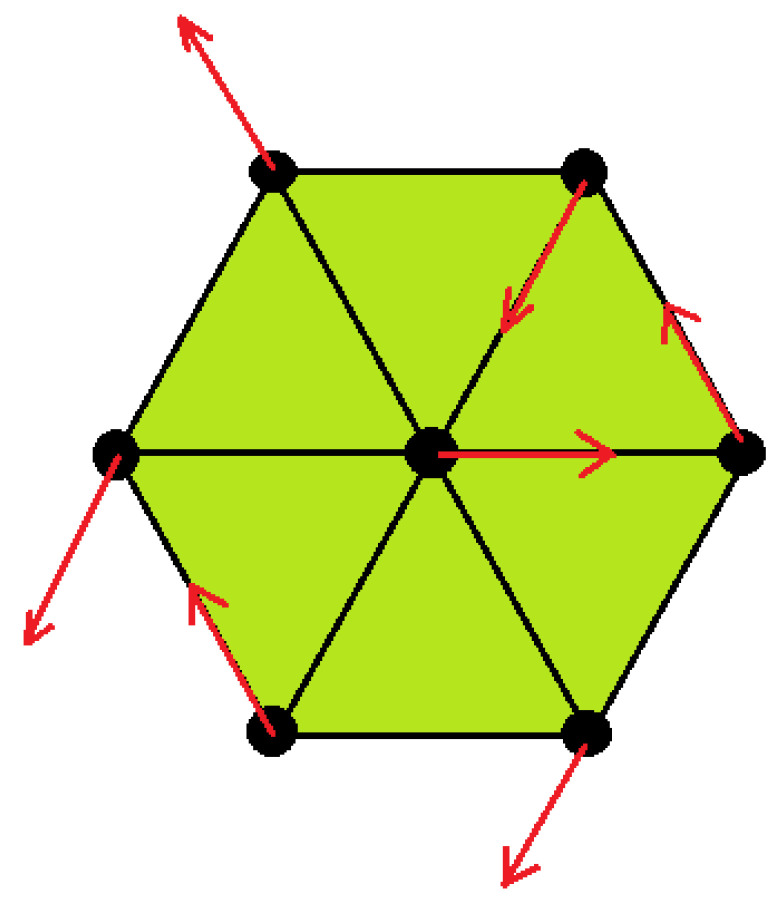
Triangular lattice: ground-state configuration. For clarity, we have taken the angle between two nearest neighbors to be equal to 120 degrees. Note that with this choice of angle the configuration is very similar to that of the antiferromagnetic triangular lattice. See text for formula of the general angle. Note also that the next-nearest neighbor (NNN) spins are parallel.

By symmetry, each spin has the same angle θ with its six NN in the xy plane. This is shown in Figure 6 with a particular choice of the angle between NN, namely 120 degrees. This angle depends on the ratio $\frac{-2J^{mf}}{J^{m}}$ as seen below. Note that the NNN spins are parallel.

For the triangular lattice, we limit ourselves to the case of NN interaction only, namely the magnetic Hamiltonian (2) without $J^{2m}$. Using this and the interface coupling (7), we write the energy of the spin $S_{i}$ as

(13) $$E_{i}=-6J^{m}S^{2}\cos\left(\theta \right)+12J^{mf}P^{z}S^{2}\sin\left(\theta \right)$$

where $\theta =|\theta_{\left(i,j\right)}|$. Care should be taken on the signs of $\sin(\theta_{\left(i,j\right)})$ when counting NN: two opposite NN have opposite signs of the spin products, and the opposite coefficients $e_{\left(i,j\right)}$. Note that the coefficient 6 of the first term is the 6 NN exchange interactions and the coefficient 12 of the second term is due to the fact that each spin has 6 interfacial coupling spin pairs with the NN polarizations in the upper ferroelectric plane, and 6 with the NN polarizations in the lower ferroelectric plane (we are in the case of a magnetic monolayer). The minimization of $E_{i}$ yields, taking $P^{z}=1$ in the ground state and S=1,

(14) $$\frac{dE_{i}}{d\theta }=0\;\;\to \;\;\theta =arctan\left(\frac{-2J^{mf}}{J^{m}}\right)$$

We see that when $J^{mf}\to 0$, one has θ→0, and when $J^{mf}\to -\infty$, one has θ→π/2 as it should be. Note that we will consider in the following $J^{mf}<0$ so as to have θ>0.

**Figure 7:**
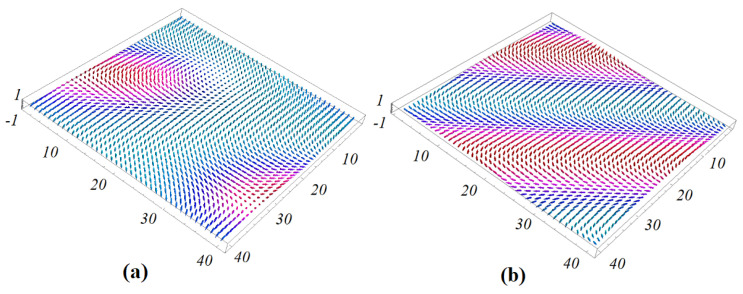
(**a**) Three-dimensional (3D) view of the ground-state (GS) configuration of the interface for H=0, $J^{m}=J^{f}=1$, $J^{mf}=-0.2$ and (**b**) $J^{mf}=-0.5$.

In the case when the superlattice has a thickness, the angle between NN in each magnetic layer is different from that of the neighboring layer. To determine the GS, we minimize the energy of each spin by using the numerical minimization “steepest descent method” as described before and in Ref. [34]. We use a sample size N×N×L. For most calculations, we select N=40 and $L=L_{m}+L_{f}=8$ using the periodic boundary conditions in the xy-plane. Exchange parameters between spins and polarizations are taken as $J^{m}=J^{f}=1$ as before. Examples of the ground state of the magnetic interface layer in zero field are shown in Figure 7, using small values of interface coupling $J^{mf}=-0.20$ and $J^{mf}=-0.5$. The case $J^{mf}=-0.20$ corresponding to small angles between spins in the xy plane so that the GS configurations have ferromagnetic and non-collinear domains as seen in Figure 7a.

For the larger value of interface magneto–electric interaction $J^{mf}=-0.5$, the GS spin configuration has a periodic non-collinear structure: each spin has the same turning angle θ with its NN in three triangular directions. We see that the periodicity of the lines connecting the NNN spins strongly depends on the value of magneto–electric interaction. When the value of the magneto–electric interaction parameter increases, the periodicity of the spin structure decreases. This dependence of the periodicity on the magneto–electric interaction can be explained by the fact that the angle θ between two NN spins is given by Equation (14) which yields the following periodicity σ (unit of lattice spacing) in each direction:

(15) $$\sigma =2\pi /\theta =2\pi /arctan(\frac{-2J^{mf}}{J^{m}})$$

The periodicity is thus shortened when $|J^{mf}|$ increases.

**Figure 8:**
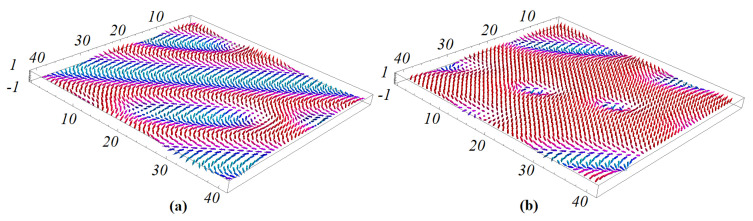
(**a**) Three-dimensional (3D) view of the ground-state configuration of the interface for H=0, $J^{m}=J^{f}=1$, $J^{mf}=-0.75$ and (**b**) $J^{mf}=-0.85$.

With increasing magneto–electric interaction parameter value, the competition between non-collinear magneto–electric interaction also increases. As a result, in the region of the magneto–electric interaction parameter [−0.75,0], a periodic non-collinear structure of the ground state is formed. Figure 8 shows an example with $J^{mf}=-0.75$ (Figure 8a) where we observe the beginning of the creation of skyrmions at the interface. Figure 8b shows an example with $J^{mf}=-0.85$ where we can observe the skyrmions at the interface and the interior magnetic layer.

**Figure 9:**
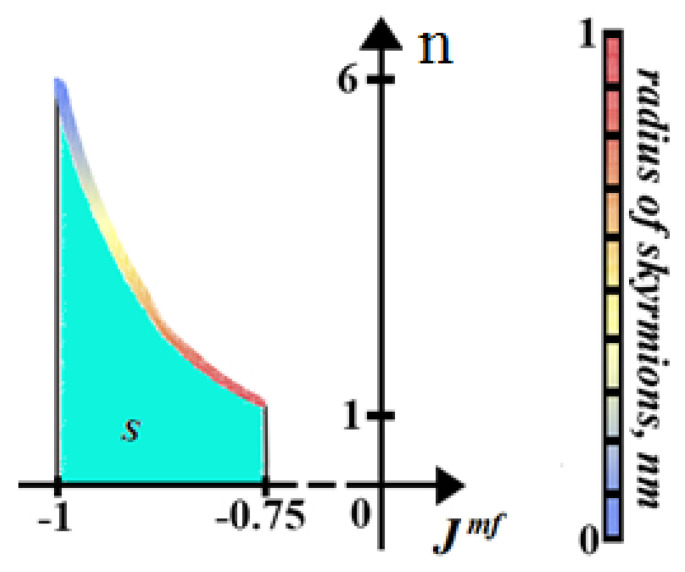
Dependence of the number of skyrmions on the interface magnetic layer *n* versus interface magneto–electric interaction parameter $J^{mf}$ in zero field. The skyrmion phase is indicated by *S*. Other parameters: H=0, $J^{m}=J^{f}=1$.

Figure 9 shows the dependence of the number of skyrmions on the interface magnetic layer *n* versus interface magneto–electric interaction parameter $J^{mf}$ in zero field. We see that, as the magneto–electric interaction becomes stronger, the number of skyrmions tends towards 6. The highest value of $J^{mf}$ where the skyrmion structure can be observed at zero magnetic field is when $J^{mf}=-1.0$. It should be noted that such skyrmions at zero field were not observed in films with a simple cubic lattice in both frustrated and unfrustrated cases. We note that skyrmions in a ferromagnetic/ferroelectric superlattices with the triangular lattice can be created in the region $J^{mf}\in \left[-1.0,-0.75\right]$ at zero field and are distributed in 3D space in the ferromagnetic film.

### 5.2. Ground State in an Applied Magnetic Field

**Figure 10:**
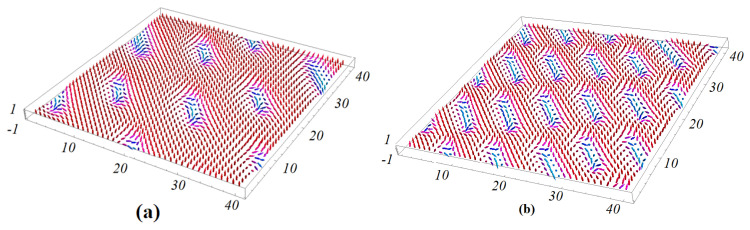
(**a**) 3D view of the GS configuration at the interface for H=0.025, $J^{m}=J^{f}=1$, $J^{mf}=-1.0$. Due to the representation scale, skyrmions seem not circular, but they are. (**b**) 3D view of the GS configuration at the interface for H=0.5, $J^{m}=J^{f}=1$, $J^{mf}=-1.75$.

We apply a magnetic field in the *z* direction perpendicular to the plane of films. Now there is a competition between ferromagnetic exchange, magneto–electric interaction between spins and polarizations at interface layers and the external magnetic field. Let us consider the critical value of the magneto–electric interaction parameter $J^{mf}=1$: at this value the skyrmion phase collapses at H=0 on the triangular lattice. We show that the GS at that point, namely, $J^{m}=1.0$, $J^{f}=1.0$, $J^{mf}=-1.0$, is very sensitive to the applied magnetic field—a clear and periodic skyrmion structure is formed for small magnitude of *H*. Figure 10a shows the case H=0.025. We see that a skyrmion lattice is formed with a perfect symmetry. The skyrmion phase for the critical value $J^{mf}=-1.0$ is stable in the region of the external magnetic field [0.01,0.24]. At H>0.24 all the spins are parallel to the *z* axis in the case $J^{mf}=-1.0$. In this model, in zero field we observe a random spatial 3D distribution of skyrmions (see Figure 8b for example) or in another model (see Figure 5b of our previous work Ref. [39]). However, in an applied field we observe a crystal structure of skyrmions when the magneto–electric coupling is strong such as the cases shown in Figure 10.

## 6. Monte Carlo Results for the Case of Triangular Lattice

Using the same method of Monte Carlo simulation and the same definitions of the magnetic and ferroelectric order parameters as for the case of simple cubic lattice, we have studied the phase transition in the magneto–ferroelectric superlattice with the triangular lattice.

**Figure 11:**
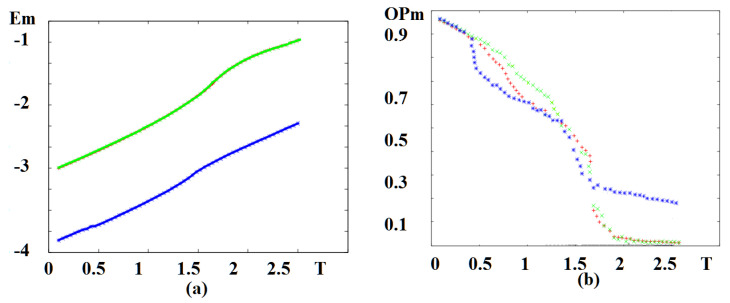
(**a**) Energy of the magnetic film on the triangular lattice versus temperature *T* for $J^{m}=J^{f}=1$, $J^{mf}=-1.0$ (red, hidden behind the green curve), coinciding with the curve for $J^{m}=J^{f}=1$, $J^{mf}=-1.75$ (green), the blue curve is for $J^{m}=J^{f}=1$, $J^{mf}=-4.25$, (**b**) order parameter of the magnetic film versus temperature with the same color code. See text for comments.

In Figure 11 we display the magnetic energy and the magnetic order parameter versus temperature *T* without an external magnetic field for various value of magneto–electric interaction. The red curve in Figure 11a corresponds to the set $J^{m}=J^{f}=1.0$, $J^{mf}=-1.0$, which coincides with the result for $J^{m}=J^{f}=1.0$, $J^{mf}=-1.75$. The change of curvature takes place at $T_{c}^{m}=\;1.75$ for both cases, meaning that the magneto–electric interaction does not affect the magnetic phase transition temperature at such a large region of $J^{mf}$ (−1.75, …, −1.0). In this region of $J^{mf}$ we do not have skyrmions at the interface without an external field. We note that in a case of magneto–electric superlattice with the simple cubic lattice, $J^{mf}$ strongly affects the critical temperature [39]. The dependence of magnetic order parameters versus *T* is shown in Figure 11b (red and green dots) confirm the phase transition from the non-collinear phase to the paramagnetic phase occurring at temperatures seen by the curvature change of the energy in Figure 11a. We display in Figure 11a also the case where $J^{m}=J^{f}=1.0$, with a strong value of $J^{mf}$: $J^{mf}=-4.25$ (blue line). As seen, such a strong magneto–electric interaction at the interface removes the phase transition: the order parameter never vanishes, as seen in Figure 11b (blue curve).

## 7. Discussion on the Applicability of the Model

Applications of skyrmions in spintronics have been largely discussed and their advantages compared to early magnetic devices such as magnetic bubbles have been pointed out in a recent review by W. Kang et al. [52]. Among the most important applications of skyrmions, let us mention skyrmion-based racetrack memory [53], skyrmion-based logic gates [54,55], skyrmion-based transistor [56,57,58] and skyrmion-based artificial synapse and neuron devices [59,60].

The manipulations with skyrmions were first demonstrated in the diatomic PdFe layer on the iridium substrate, and the importance of this achievement for the technology of information storing is difficult to overestimate: it makes possible to write and read the individual skyrmions using a spin-polarized tunneling current [7]. In Ref. [61], the possibility of the nucleation of skyrmions by the electric field by means of an inhomogeneous magneto–electric effect was established.

The above mentioned applications of skyrmions have motivated the present work. Our model is interesting when the magneto–electric interaction is large, in the order of the magnetic interaction. Fortunately, this condition is realizable using the magneto–electric coupling in superlattices. Let us cite a number of works showing strong and very strong magneto–electric (ME) interactions to justify the applicability of our model. Values of ME interactions depend on the materials, but they are of the order of the ferromagnetic interaction or even larger as found in a lot of experiments among which we mention below. We note that ideal multiferroic memory cell could offer the possibility to electrically write the magnetic state, which then can be read out in a non-destructive manner. Such a multiferroic memory concept combines the high speed of existing electronics with the remnant properties of magnetism and thus joins the best aspects of existing ferroelectric and magnetic data storage memories. The realization of such devices requires an electrical control of magnetism, i.e., a strong magneto–electric coupling between the magnetic moments and the electric polarizations. Magneto–electric materials are one example of "smart" materials in which the coupling of such different physical quantities produces new phenomena, which are more than just the sum of the original ones, possessing, e.g., sensing and actuating functions in the same compound. The magnitude of the novel coupling phenomena between dielectric and magnetic properties is bounded by the product of the permittivity and the permeability in case of magneto–electric materials. Thus, compounds which easily polarize in response of an electric and magnetic field may exhibit large magneto–electric effects. This requirement is usually fulfilled by multiferroic materials (see Ref. [62] and references cited therein). We mention a few experimental works among the numerous experiments showing large magneto–electric couplings with comments on some works [63,64,65,66,67,68].

## 8. Conclusions

We have studied the phase transition and ground-state configurations in superlattices formed by alternate magnetic and ferroelectric films by the use of Monte Carlo simulation and the steepest descent method. We have taken into account a DM interaction at the interface and the frustration due to the NNN interactions. The crystal structure is the simple cubic lattice, the magnetic spins are the Heisenberg model and the polarizations are assumed to be of an Ising-like model. The skyrmion crystal is shown to be formed under an applied magnetic field and is stable at finite temperatures. This may have interesting applications in transport by skyrmions.

In the second part, we have considered the magnetic and ferroelectric films of triangular lattice with respectively Heisenberg spins and Ising-like polarizations. We found the formation of a stable skyrmions in the ground state of ferromagnetic/ferroelectric superlattices even at zero applied magnetic field in a region of interface coupling $J^{mf}\in \left[-1.0,-0.75\right]$. In the ferromagnetic film the skyrmions are distributed in 3D space. Very strong magneto–electric interactions at the interface leads to the disappearance of the magnetic phase transition, unlike the case of simple cubic lattice where we observed very strong first-order phase transitions at large values of the interface magneto–electric interaction. The existence of skyrmions at the magneto–ferroelectric interface in the ground state at zero magnetic field is very interesting and may have practical applications in digital technologies and spintronics. We found the formation of a perfect skyrmions lattice at acceptable values of magneto–electric interaction in a large region of the external magnetic field.
